# Retention of bimanual performance following hand arm bimanual intensive therapy in children with unilateral cerebral palsy: A six-month longitudinal study

**DOI:** 10.1371/journal.pone.0313018

**Published:** 2024-12-31

**Authors:** Shailesh S. Gardas, Christine Lysaght, Charity Patterson, Swati M. Surkar

**Affiliations:** 1 Dept of Physical Therapy, East Carolina University, Greenville, NC, United States of America; 2 Dept of Physical Therapy, University of Pittsburg, Pittsburgh, PA, United States of America; University of Illinois Urbana-Champaign, UNITED STATES OF AMERICA

## Abstract

Hand-arm bimanual intensive therapy (HABIT) enhances upper extremity (UE) function and bimanual coordination in children with unilateral cerebral palsy (UCP). Previous studies assessed immediate improvements in UE function using clinical and self-reported measures, which may not accurately reflect real-world UE performance and their long-term retention effects. Therefore, this study aims to investigate the retention of real-world bimanual performance gains over time following HABIT in children with UCP. Thirty children with UCP, age 6–16 years underwent HABIT (6 hours/day for 5 days). Bimanual performance was assessed using GT9X Link accelerometers, worn on bilateral wrists for 3 days pre-, post-, 3-, and 6-month of HABIT. Accelerometer-derived variables–use ratio (UR), magnitude ratio (MR), bilateral magnitude (BM), median acceleration (MA), and acceleration variability (AV)–quantified bimanual performance during real-world activities. UE function was measured with standardized assessments. A mixed model analysis with repeated measures and paired t-tests analyzed the differences real-world bimanual performance and UE function respectively. There was a significant main effect of time in UR (F = 2.72, p = 0.05), BM (F = 4.36, p = 0.007), and MA (F = 3.68, p = 0.016). Post-hoc analysis (mean differences, 95% confidence interval [CI]) revealed improvements immediately post- compared to pre-HABIT in BM (14.99, 4.35–25.63) and MA (7.46, 2.55–12.36). However, subsequent assessments at 3- and 6-months displayed a regression in these gains, suggesting a lack of retention. A decline was observed at 3 months) and 6 months (BM; 16.94, 6.3–27.4, MA; 6.51, 1.61–11.41) in BM and MA compared to post-HABIT. UE capacity measures also showed improvements (p < 0.05) post-HABIT. Although HABIT initially may enhance performance of real-world bimanual tasks, its benefits diminish within six months, suggesting a need for repeating HABIT every 3–6 months to retain long-term improvements.

## Introduction

Cerebral Palsy (CP) is the leading cause of physical disability in children, affecting approximately 1 in 500 live births [1, 2]. Around 35% of children with CP have unilateral CP (UCP), which affects one half of their body, leading to substantial limitations in bimanual hand use further impacting their daily activities and quality of life [3]. As a result, recent research has focused on therapeutic interventions to improve the upper extremity (UE) function and enhance bimanual coordination [4–8]. Evidence suggests that intensive interventions like hand-arm bimanual intensive therapy (HABIT) and constraint-induced movement therapy (CIMT) are effective in enhancing bimanual coordination and the affected hand function in children with UCP [9]. However, most studies on intensive training protocols have only examined immediate improvements in UE function following therapy [10–12]. There is limited research examining the long-term retention of treatment benefits in children with UCP [13, 14]. Additionally, these studies assessed bimanual function using standardized, lab-based assessments that measure hand capacity [10–12]. Capacity, as per the International Classification of Functioning, Disability and Health (ICF), is what a child does in a controlled lab environment, whereas performance is what a child actually does in the outside real-world scenario [15]. None of the prior studies have assessed retention of real-world bimanual performance that reflect activities performed in a real-world context.

The ultimate goal of rehabilitation is to enhance a child’s ability to perform daily activities necessary for independent living [16]. Intensive therapies like CIMT and HABIT encourage the practice of real-life activities through self-generated movements and aim to achieve real-world activity goals that are collaboratively set by the child and parent [17]. Therefore, the assessments following these intensive therapies should capture the change in real-world bimanual activities using objective tools such as body worn sensors or accelerometers that track data for several hours to days. The data gathered from accelerometers offer valuable insights into changes in bimanual movement characteristics in the real-world contexts following intensive therapies in children with UCP [18]. However, majority of previous studies assessing the affected hand function and bimanual coordination post-CIMT and HABIT have relied on standardized assessments such as the Assisting Hand Assessment, box and block test, Jebsen hand function test, and 9-hole peg test [10] that primarily evaluate hand function in controlled laboratory settings, focusing on the hand capacity [15]. Furthermore, a few studies investigating the long-term retention of upper extremity performance have used subjective measures such as the Canadian Occupational Performance Measure (COPM) and the Pediatric Evaluation of Disability Inventory (PEDI) that are susceptible to social desirability and recall biases and may not accurately measure the real-world performance [13, 19–22]. Therefore, there is an urgent need to employ an objective tool that can overcome these limitations and accurately quantify retention of real-world activities, effectively reflecting bimanual use in everyday life in a convenient manner.

Previously, real-world bimanual activities have been measured among individuals post-stroke [23, 24] and children with CP [18, 25] using accelerometers. Accelerometers provide a valid and reliable assessment of direct UE performance, enabling the evaluation of various bimanual movement characteristics [25]. They quantify the contribution of the more affected UE compared to the less affected UE throughout the bimanual activities of daily living, measured through metrics like use ratio and magnitude ratio [26, 27]. Additionally, accelerometers can also be used to isolate and measure the average accelerations and movement variability in the more affected UE through metrics such as median acceleration and acceleration variability. Our recent research exhibited substantial gains in real-world bimanual performance immediately following 30 hours of HABIT in children with UCP [18]. Yet, the long-term retention of these real-world gains following intensive therapies assessed using body-worn sensors remains uncertain [13, 14, 28, 29]. Therefore, it is crucial to objectively evaluate the retention of bimanual performance after HABIT over time in real-world settings. Furthermore, given the substantial time, effort, and economic costs associated with intensive therapies, clinicians expect to achieve long-lasting improvements in hand function following these interventions. Gaining a deeper understanding of the trajectory of UE functional outcomes after intensive therapies can help clinicians in determining when treatment benefits start to decline. This knowledge can aid therapists estimate when to reintervene, ultimately improving the child’s functioning and reducing the burden on stakeholders.

Therefore, the primary goal of this novel study was to investigate retention of real-world bimanual performance over time following HABIT in children with UCP using accelerometers to gain valuable insights into their daily activity and participation over time. Our hypothesis was that performance gains immediately after HABIT will gradually diminish within a few months. The study findings could offer valuable information to clinicians regarding the temporal patterns of decline in intensive therapy’s benefits; therefore, guiding timely re-intervention to prevent further deterioration and facilitate improvements in hand function.

## Materials and methods

### Trial design

This study is an ancillary analysis of a clinical trial (NCT05355883). It was a longitudinal, prospective study conducted in the Pediatric Assessment and Research Laboratory (PeARL) at East Carolina University (ECU), NC. The University and Medical Center Institutional Review Board, ECU approved the study. We obtained written informed parental consent and child assent.

### Participants

In this study, we included children with UCP, within the age group 6–16 years, Manual Ability Classification system (MACS) levels I–III, and children who can complete a stack of three cups within one minute. Children with neuromotor disabilities other than UCP, cognitive and communication deficits, uncorrected vision, contractures (wrists, and hands), a history of botulinum neurotoxin injections on the affected UE, history of recent UE surgery, and history of intensive therapy (HABIT or CIMT) in the past 6 months were excluded from the study.

### Procedures

Schematics of the study timeline including the HABIT and assessments schedule

#### Hand Arm Bimanual Intensive Therapy (HABIT) intervention protocol

HABIT is a well-established intervention that improves the affected upper extremity (UE) function and bimanual coordination in children with UCP. The details of the HABIT intervention have been reported in our previous published work [11, 30]. We delivered HABIT in a camp-based setting involving task-specific, structured, bimanual activities. The dose of HABIT was 6 hours per day, for 5 consecutive days, totaling 30 hours. Children were engaged in age-appropriate bimanual gross and fine motor activities in a playful context. The participating children had varied levels of baseline hand function; hence, individualized therapy goals were formulated based on their pre-training AHA performance. Each child was assigned with four trained interventionists (physical or occupational therapy students) under the supervision of licensed physical therapists to ensure fidelity of therapy. Interventionists progressively increased the complexity of bimanual activities and graded the tasks demands based on the performance to allow the child to complete the tasks successfully. Throughout the camp, children were encouraged to use the affected and the less affected UE in a coordinated manner. Positive reinforcement and knowledge of performance were provided to motivate and reinforce desired goal-directed activities. Emphasis was placed on different roles of the affected UE, such as stabilizer, manipulator, and assistor depending on the child’s ability and task goal. Sessions comprised of whole-task and part-task practice. Activities performed by the children were documented by the interventionists.

#### Accelerometer data acquisition

We used bilateral wrist-worn accelerometers (ActiGraph GTX9 Link, Pensacola, FL, USA) to assess real-world bimanual performance. Actigraph GT9X Link measures accelerations in activity counts along three axes, where one count equals 0.001664g. The accelerometer data obtained was sampled at 30 Hz, and activity counts were binned into 1-second epochs in ActiLifeTM 6 software [26]. Finally, the data was processed in MATLAB (Mathworks Inc, Natick, MA, USA) using custom-written code developed by Lang et al. [26].

In this longitudinal study, we evaluated immediate gains in bimanual performance by analyzing changes in accelerometer-derived variables pre- and post-HABIT. To obtain pre- and post-HABIT accelerometer data, children were required to wear the accelerometers for 3 days before and 3 days immediately after 30 hours of HABIT. The three days wearing time was chosen since it produces a reliable estimate of performance in children with CP [31]. Long-term retention in bimanual performance was assessed by evaluating the changes in accelerometer derived variables over different time points from HABIT: pre-, post-, 3-month, and 6-month. To acquire the 3- and 6-month follow-up data, a pair of accelerometers were sent to the children’s respective mailing addresses. Clear written instructions for wearing and using the accelerometers were provided in the package for both children and parents. They were specifically instructed to wear the accelerometers during waking hours while performing their daily routines, and to remove them during extended water-related activities like bathing or swimming. Parents were also informed to return the accelerometers back to the laboratory address once the stated wearing time was completed.

#### Outcome measures

**Fig 1:**
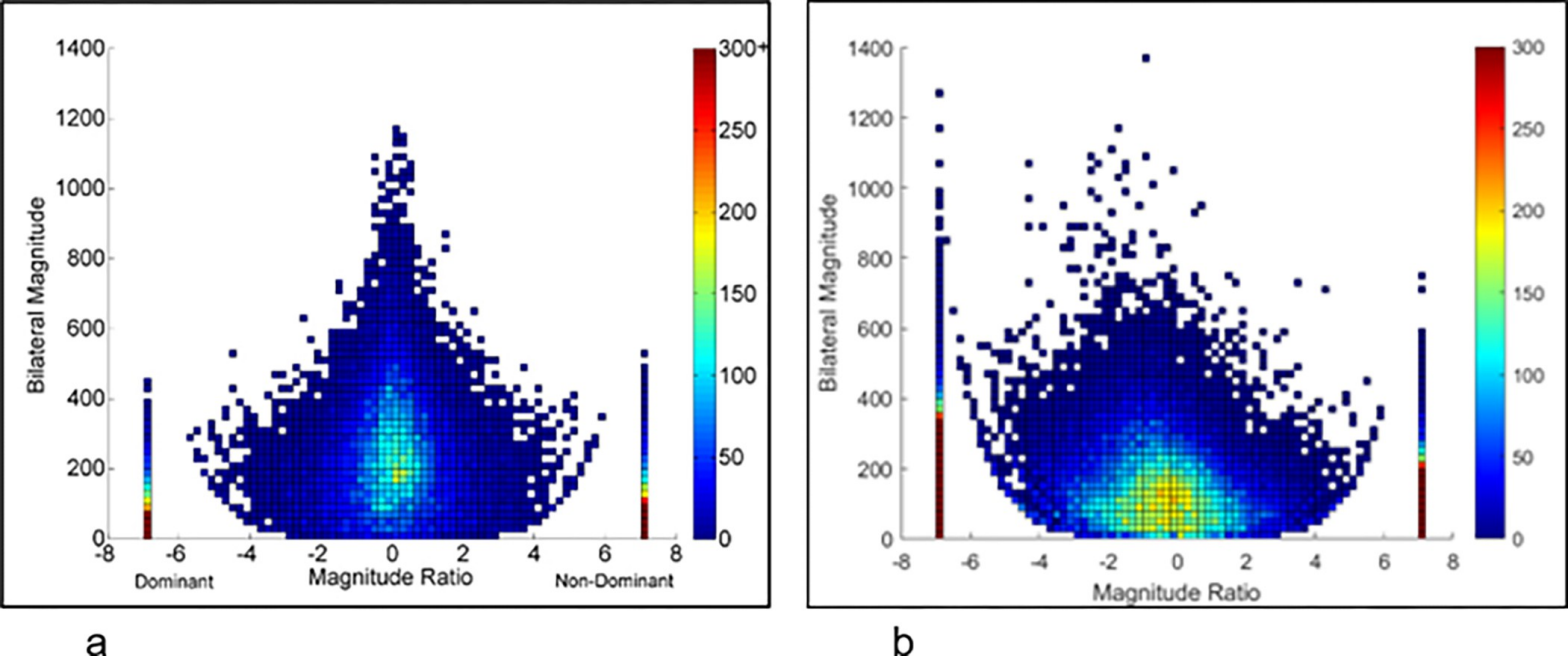
Representative examples of density plots. Graphical representations of accelerometer data of both the upper extremities (UE) of (a) a typically developing child and (b) an age-matched child with right sided UCP pre-HABIT. The density plots are obtained by plotting the accelerations on a second-by-second basis recorded over a total wear time of 3 days pre-HABIT. The x-axis (magnitude ratio) indicates the contribution of each extremity to the task, and the y-axis indicates the overall intensity of movement. The right and left halves of the plots represent right and left UE use, respectively. In Fig 1b, the right half is more affected, and left is less affected UE of the child with UCP. The dots seen in the graphs represent the counts (number) of movements (accelerations) performed by each UE. The colors in the large color bar scale on the right of the plot indicate the frequency of movements, where brighter colors represent greater frequencies and vice versa. In the above representative examples, Fig 1a appears noticeably symmetrical as compared to Fig 1b. It indicates that the typically developing child used both UEs equally in terms of hours, whereas the child with UCP used the affected UE considerably less (right half of Fig 1b) relative to left, resulting in an asymmetric density plot, which is termed as use ratio, a measure of duration of affected relative to less affected UE. Magnitude ratio is the contribution of the affected relative to the less affected UE in terms of intensity of movements. The dots seen in both the halves of Fig 1a appear at similar heights suggesting symmetrical magnitude of both UE movements in a typically developing child. Whereas, in Fig 1b, the dots representing right (affected) UE are distinctly lower as compared to less affected UE, resulting in MR value moving towards negative side. Bilateral magnitude is the overall intensity of both UEs. The overall height of Fig 1a is taller relative to Fig 1b, indicating lower intensity of movements in the child with UCP.

*Upper extremity performance outcomes*: *Accelerometer derived variables*. Upper extremity performance was assessed using the accelerometer derived variables: 1) use ratio (UR), 2) magnitude ratio (MR), 3) bilateral magnitude (BM), 4) median acceleration (MA), and 5) acceleration variability (AV). Fig 1 describes the specifics of these variables using density plots.

1. Use ratio measures the total duration of the affected UE activity relative to the less affected UE throughout the wearing period. UR has a value ranging from 0 to 1. The UR close to or equal to 1 indicates symmetric use of both UEs in daily life. The UR closer to zero indicates a higher use of less affected UE [26].

$$Use\;ratio=\frac{Hours\;of\;affected\;upper\;extremity\;use}{Hours\;of\;less\;affected\;upper\;extremity\;use}$$

**Fig 2:**
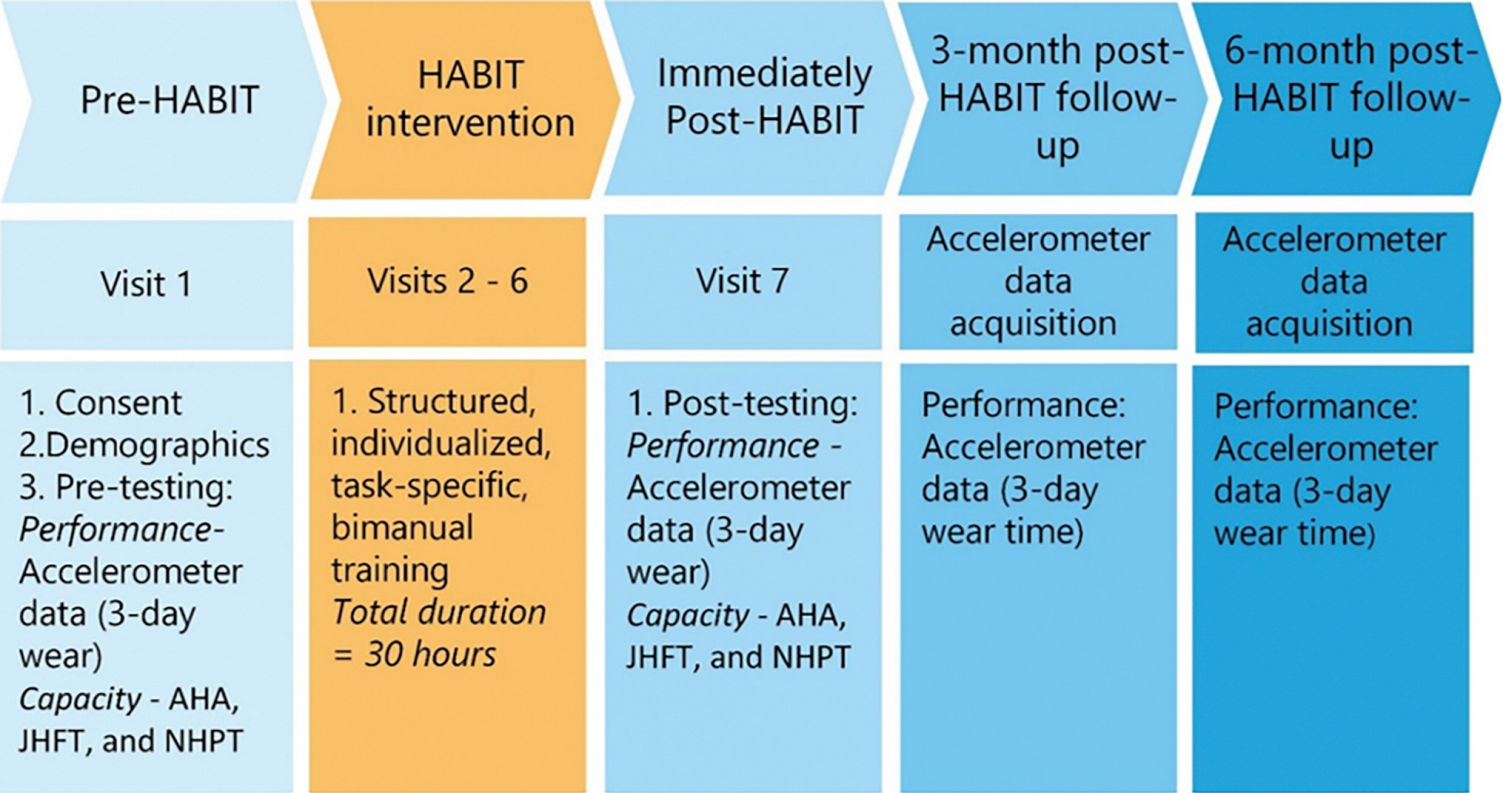
Schematics of the study timeline. Abbreviations: AHA = Assisting hand assessments, JHFT = Jebsen hand function test, NHPT = Nine-hole peg test.

2. Magnitude ratio measures the contribution of the affected UE relative to less affected UE during a bimanual activity in terms of magnitude/intensity. In the density plots of Fig 2, MR is plotted on the x-axis with a value ranging from -7 to +7. A value closer to 0 indicates equal contributions from both UEs; positive values indicate greater movement intensity of the affected UE, and negative values indicate greater intensity of the less affected UE [26].

$$Magnitude\;ratio=\frac{Magnitude\;of\;affected\;upper\;etremity\;use}{Magnitude\;of\;less\;affected\;upper\;extremity\;use\;}$$

3. Bilateral magnitude reflects the overall intensity of activity across both UEs and is calculated by summating the smoothed vector magnitudes of both UEs for each second of activity. BM is plotted on the y- axis of the density plot and a value of zero indicates no activity and increasing value indicates greater intensity of bilateral UE activity [26].

$$Bilateral\;magnitude=Magnitude\;of\;(more\;affected+less\;affected\;{UE}^{'}s)$$

4. Median acceleration and acceleration variability reflect the performance characteristics of the more affected UE. MA represents the acceleration of the affected UE magnitude over the entire wear time. AV indicates the distance of the affected UE acceleration from the average acceleration over the monitoring period. A higher score for both these variables indicates better overall UE movements [26].

*Upper extremity capacity measures—Body function and activity domains of ICF*. Standardized clinical tests such as Assisting Hand Assessment (AHA), Jebsen Hand Function test (JHFT), and Nine Hole Peg test (NHPT) were used to measure changes in the UE capacity pre- and immediately post-HABIT [15]. The AHA measures the affected hand function and bimanual coordination in children with UCP (reliability: interrater = 0.98, intra-rater = 0.99) [15, 32, 33]. An improvement of 5 units is considered clinically meaningful [33]. JHFT (reliability; interrater = 0.94, test-retest = 0.91) [34] and NHPT (reliability; interrater = 0.99, test-retest = 0.81) [35] measures unimanual dexterity and speed.

### Statistical analysis

Statistical analyses were performed using SPSS (version 28.0; IBM Corporation, Armonk, New York). Capacity outcomes were assessed for normality using the Shapiro–Wilk test. To determine changes in the upper extremity capacity measures immediately post-HABIT, we used a paired t-test for AHA and NHPT, as the data was normally distributed. We employed the Wilcoxon Signed Rank test for JHFT, as their data violated the assumption of normality. Results are presented as a mean ± standard error of the mean. The significance level was set at ≤ 0.05 for the paired t-test analysis. A linear mixed model for repeated measures was conducted to assess long-term retention in the accelerometer derived variables across the different testing time-points i.e., pre-, post-, 3-month, and 6-month after HABIT. To test the main effect of time, the significance level was set at α ≤ 0.05. The model included time-points as the fixed effect and random intercept of subjects to account for within-subject correlations. We used the Akaike information criterion (AIC) to assist with selecting the appropriate statistical model. A lower AIC value indicates better quality of fit; thus, we chose the model that demonstrated the minimum AIC. A Kenward-roger approximation was used to compute the degrees of freedom. Post hoc tests with Bonferroni multiple corrections were used for pairwise comparisons between time points. Normality assumptions for linear mixed model were tested by visualizing the normal Q-Q plot and then conducting the Shapiro-Wilk test on the residuals. The significance level was set at α ≤ 0.01 to account for multiple comparisons.

## Results

**Fig 3:**
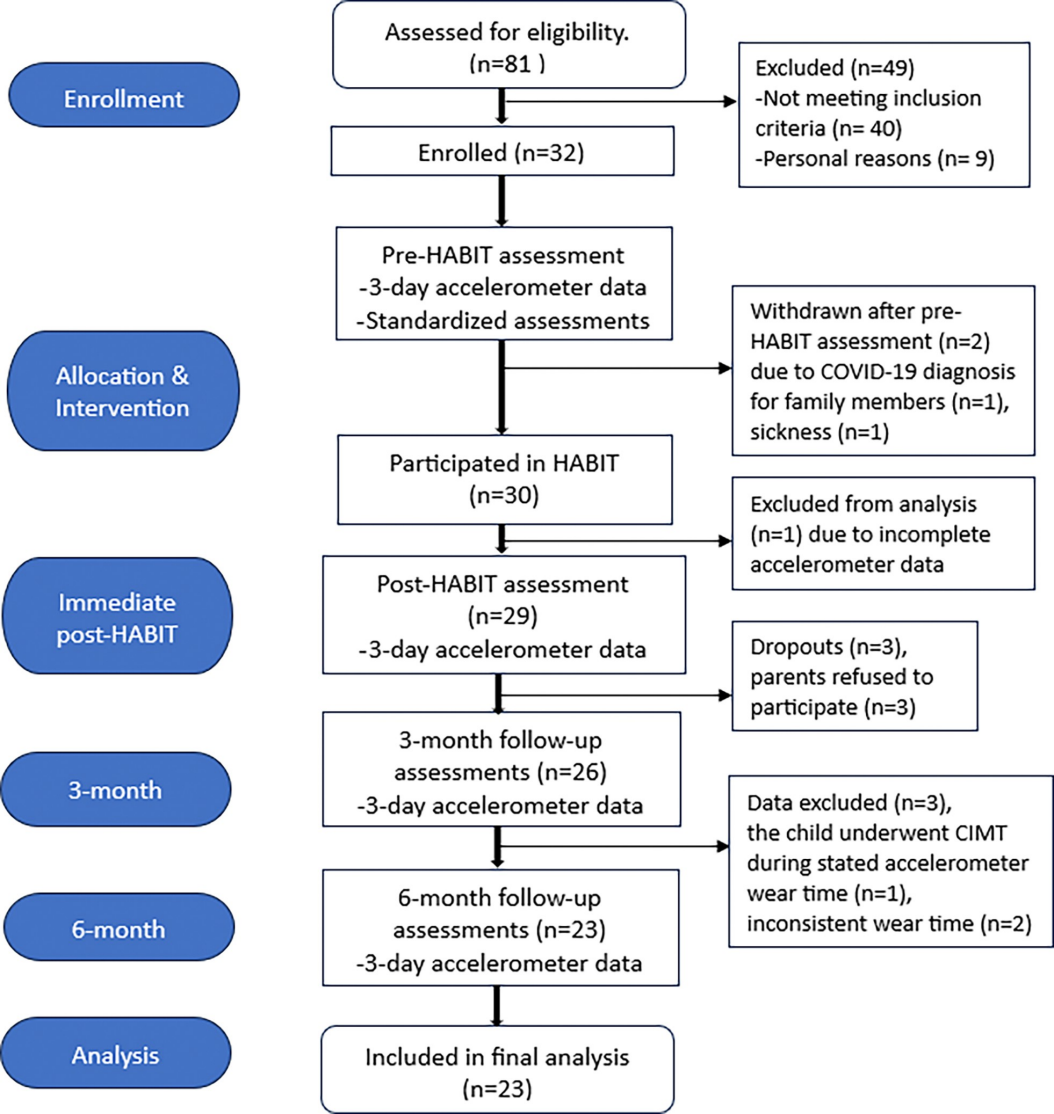
CONSORT diagram. A total of 81 participants were assessed for eligibility and 30 participants eventually participated and completed the HABIT protocol. Twenty-nine participants were included in the immediate post-HABIT analysis. Six participants were excluded during the follow-up longitudinal accelerometer assessments. Twenty-three participants were included in the final analysis.

Thirty-two children with UCP, age 6–16 years and Manual Ability Classification system levels I–III participated in this study. Fig 3 illustrates the CONSORT diagram, detailing how participants progressed through the study, including withdrawals, and their inclusion in the analysis. Table 1 displays the details of the participant demographics. Power analysis was conducted in G*Power [36] based on the primary outcome measure, use ratio (pre-HABIT = 0.73 ± 0.08, post-HABIT = 0.78 ± 0.11, effect size = 0.51), as reported in our previous study. (18) Given the absence of longitudinal studies assessing bimanual performance using accelerometer-based metrics, we performed a post-hoc power analysis to evaluate the retention effects on use ratio. Utilizing a repeated measures design for the within-subjects factor, to detect a medium effect size (f = 0.25) at a significance level of 0.05, a total of 23 participants provided 89.2% power to the study for detecting long-term retention effects.

**Table 1:** Demographic characteristics of the participants.

Characteristics	Participants
	Children with Unilateral Cerebral Palsy (n = 23)
Sex, n (%)	
Male	16 (70)
Females	7 (30)
Age, mean (SD)	11.68 (3.26) years
Side of hemiplegia, n (%)	
Left	11 (48)
Right	12 (52)
Race, n (%)	
White	20 (87)
African American	-
Asian	3 (13)
Multiracial	-
MACS Level, n (%)	
I	2 (9)
II	10 (43)
III	11 (48)

Demographics of the participants during pre- and post-HABIT time points. MACS indicates Manual ability classification system.

### Long-term retention of bimanual performance during the follow-up assessments

The mixed model analysis with random intercept revealed significant between-subject’s variability in all accelerometer-derived variables. Table 2 provides a summary of estimated subject and residual variances for accelerometer variables. Intra-class Correlation coefficient (ρ) values were as follows: UR = 0.75, MR = 0.51, BM = 0.56, MA = 0.71, and AV = 0.62. The higher ICC values indicate homogeneity of responses in all the accelerometer variables amongst participants justifying the use of mixed model analysis. The differences in mean scores for all accelerometer variables across all four time points are detailed below.

**Table 2:** Estimates of covariance parameters.

Accelerometer variables	Residual	Intercept (subject)	ICC (β)
Use ratio	0.003	0.01	0.75
Magnitude Ratio	0.83	0.91	0.51
Bilateral Magnitude	326.4	360.44	0.56
Median Acceleration	69.24	159.71	0.71
Acceleration Variability	95.93	166.81	0.62

ICC = Intraclass correlation coefficient

#### Use ratio

**Fig 4:**
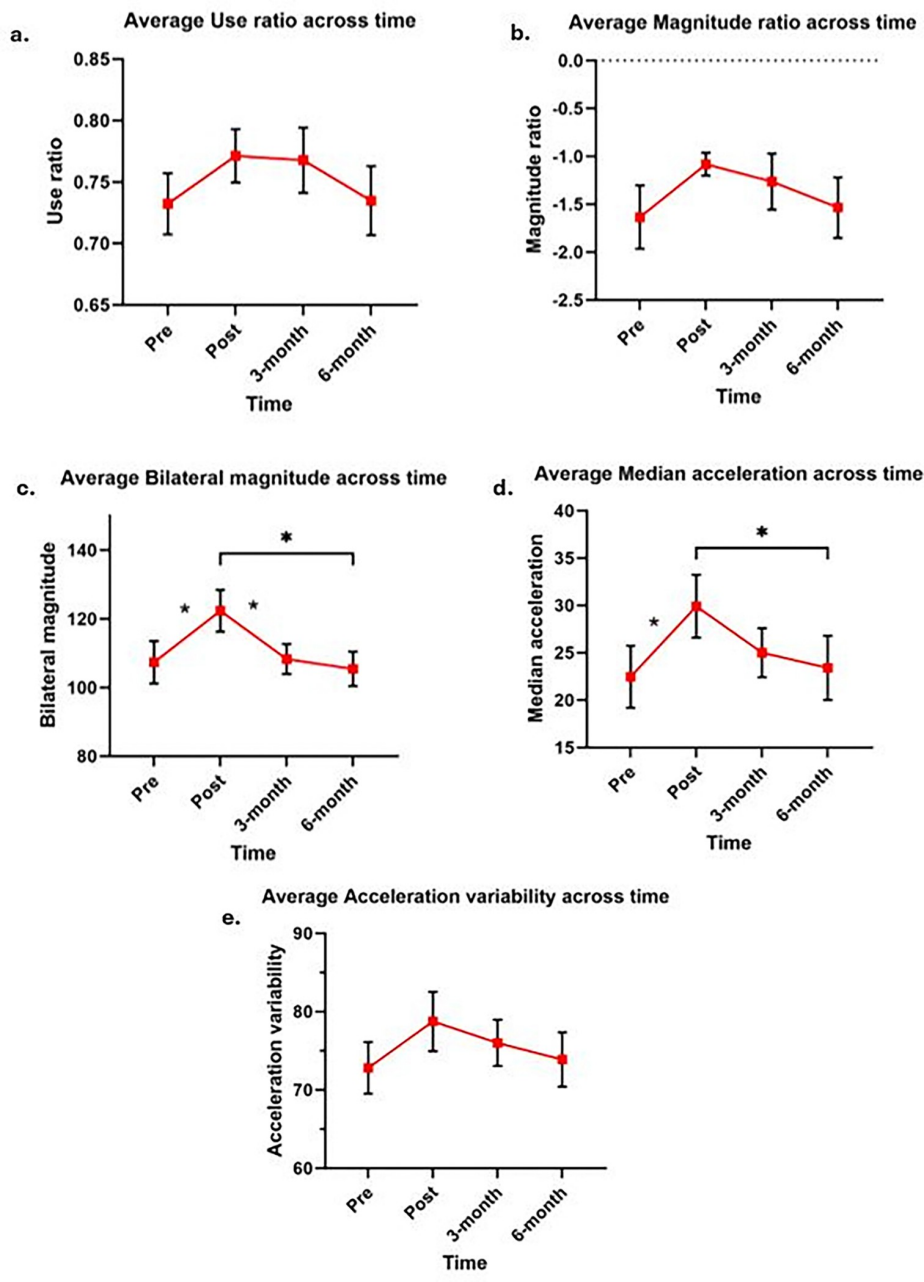
Differences in mean scores of upper extremity performance in all five accelerometer-derived variables across four time points- pre-HABIT, post-HABIT, 3-month, and 6-month post-HABIT. Values reported are means ± SEM as determined by distribution of data during each assessment time point. (a) Use ratio, (b) Magnitude ratio, (c) Bilateral magnitude, (d) Median acceleration, and (e) Acceleration variability. Pre-HABIT refers to baseline assessment and post-HABIT refers to assessments immediately within a week following HABIT. 3-month and 6-month time points refers to accelerometer data acquired 3- and 6-months following HABIT to assess long term retention of bimanual performance. The trends seen in the line graphs above indicate a decrease in all the accelerometer derived variables at 3-month time point (except use ratio) suggesting decrease in bimanual performance. Furthermore, all the accelerometer variables nearly returned to baseline or pre-HABIT levels at the 6-month follow up assessments. *denote significance with p value ≤ 0.01 on Bonferroni pairwise comparison between the study time points.

Longitudinal analysis indicated a main effect of time for the use ratio (F = 2.72, p = 0.047). Post-hoc analysis revealed trends of improvements in UR (pre = 0.73 ± 0.025, post = 0.77 ± 0.025, Fig 4A) immediately post-HABIT. No considerable differences were detected between mean scores of UR post-training (mean = 0.77, SE = 0.025) and at 3-months (mean = 0.76, SE = 0.025). However, at 6 months (mean = 0.74, SE = 0.025), the UR declined, nearly returning to the pre-training levels. These findings suggest that the HABIT resulted in gains in contributions of the more affected UE in terms of hours of use, immediately post-HABIT and these improvements were retained for 3 months following HABIT reflecting retention of performance gains. However, subsequently at 6 months, these improvements were lost and returned to baseline levels.

#### Magnitude ratio

There was no main effect of time detected for the magnitude ratio (F = 1.77, p = 0.16). The trends in longitudinal assessments revealed improvements in the mean scores of MR immediately post-HABIT (pre = -1.6 ± 0.28, post = -1.1 ± 0.28, Fig 4B, p = 0.04). Means at 3-month (mean = -1.26, SE = 0.28, p = 0.17) and 6-month (mean = -1.54, SE = 0.28, p = 0.71) were similar to baseline. Overall, the findings suggest marginal improvements in the contributions of the more affected UE in terms of magnitude in real-world performance immediately post-HABIT, followed by an absence of retention at the follow-up time-points.

#### Bilateral magnitude

There was a main effect of time for bilateral magnitude (F = 4.36, p = 0.007). Post-hoc analysis showed that mean BM scores at post-training (mean = 122.3, SE = 5.5) were higher from those at pre-training (mean = 107.3, SE = 5.5, p = 0.006, Fig 4C, Table 3), 3-month (mean = 107.3, SE = 5.5, p = 0.007) and the 6-month time-points (mean = 105.4, SE = 5.5, p = 0.002). These findings indicate that HABIT resulted in immediate improvements in the total magnitude of movements performed by bilateral UEs, but the gains were dramatically lost at 3 month’s follow-up assessments. The overall magnitude of movements nearly returned to the baseline at 6-month time-point suggesting no retention of real-world performance.

**Table 3:** Pairwise comparison of the bilateral magnitude and median acceleration between different assessment time points.

Bonferroni Pairwise comparison								
Accelerometer Variables	Mean	vs	Mean	Significance	Mean difference	Standard error	Lower Bound	Upper Bound
Bilateral magnitude	Pre	vs	Post	0.006*	14.99	5.33	4.35	25.63
	Post	vs	3-month	0.007*	15.05	5.40	4.26	25.84
	Post	vs	6-month	0.002*	16.94	5.32	6.30	27.58
Median acceleration	Pre	vs	Post	0.003*	7.76	2.45	2.55	12.36
	Post	vs	6-month	0.01*	6.51	2.45	1.61	11.41

Post hoc analysis results with Bonferroni multiple comparisons between different time points

* indicates significant p value at α = 0.01.

#### Median acceleration

There was a main effect of time for median acceleration (F = 3.68, p = 0.016). Post-hoc analysis, employing Bonferroni multiple comparisons, showed that mean MA scores at post-training (mean = 29.9, SE = 3.2, Fig 4D, Table 3) were higher from those at pre-training (mean = 22.5, SE = 3.2, p = 0.003), and 6-month (mean = 23.4, SE = 3.2, p = 0.01). These findings suggest that HABIT resulted in gains in the accelerations of the more affected UE in the real-world situations, immediately post-HABIT. However, these gains were lost at the 3-month time-point, almost returning to baseline values at 6 months, suggesting lack of retention.

#### Acceleration variability

Longitudinal analysis showed no differences by time for the acceleration variability (F = 1.61, p = 0.19). The longitudinal assessments revealed a trend in improvement in the mean scores of AV immediately post-HABIT (pre = 72.8 ± 3.4, post = 78.8 ± 3.4, Fig 4E, p = 0.04). These findings indicate enhanced movement variability of the more affected UE in the outside world immediately post-HABIT. However, a declining trend is observed following the immediate gains at 3-month, nearly returning to baseline at 6 months post-HABIT, reflecting lack of retention.

### Immediate gains in upper extremity capacity post-HABIT

**Fig 5:**
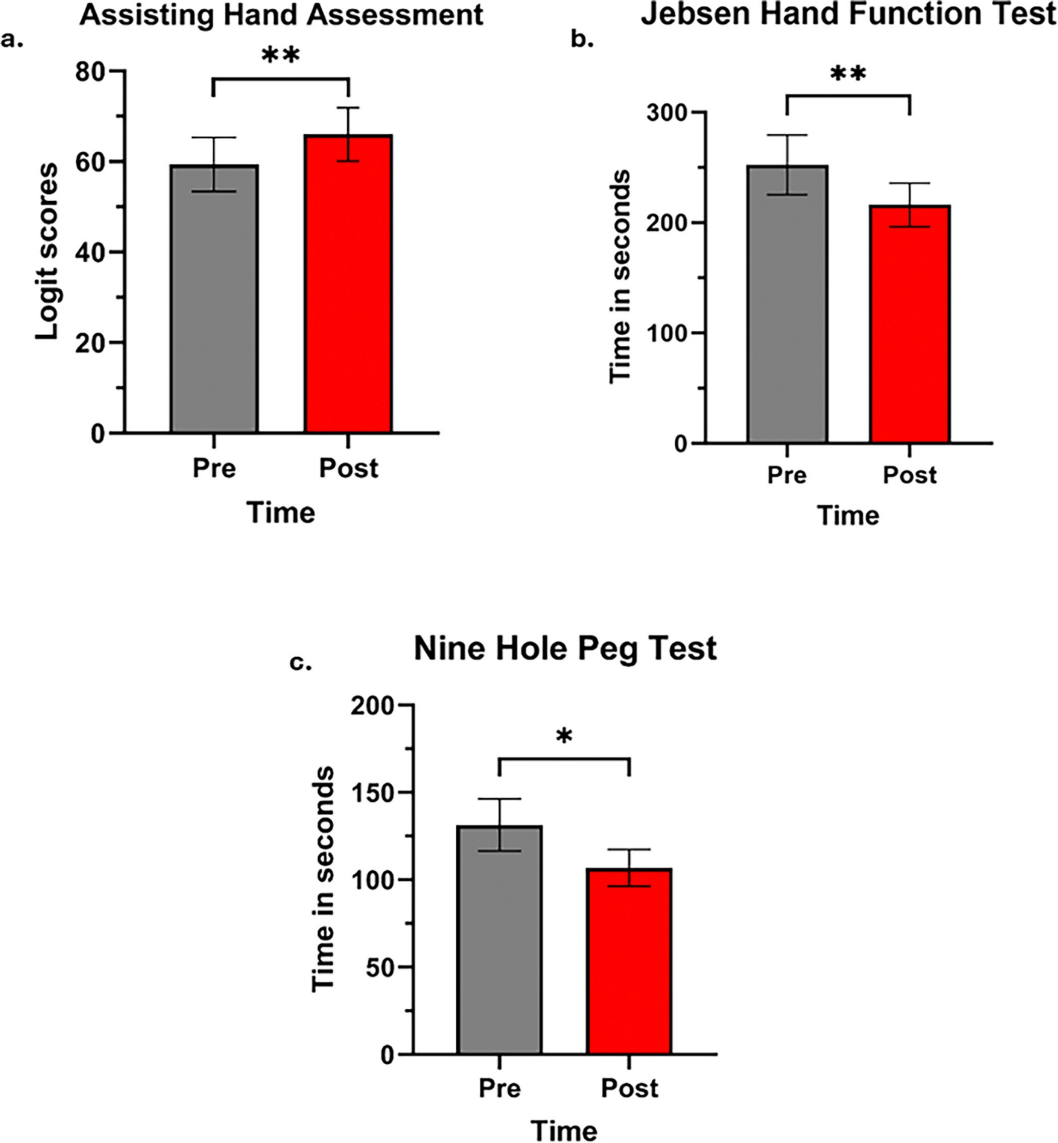
Comparison of differences in the mean scores of capacity measures pre- and post-HABIT training. Values reported are means ± SEM as determined by distribution of data during each assessment time point. Pre-training refers to baseline assessment and post-training refers to assessment within one week following HABIT. There were significant changes seen in mean scores of (a) Assisting Hand Assessment, (b) Jebsen Hand function test (more affected UE), and (c) Nine Hole Peg test (more affected UE) from pre- to post-training. Children demonstrated greater change in the AHA indicating greater function of the affected arm and bimanual coordination, faster speeds in JHFT and NHPT during post-training assessment compared to baseline. *denotes p < 0.05, ** denotes p < 0.005.

There were also improvements in the mean scores of the AHA (p = 0.001, Fig 5A), the JHFT (p = 0.004, Fig 5B), and the NHPT (p = 0.02, Fig 5C) from pre- to post-HABIT, reflecting gains in capacity. These findings indicate that immediately post-HABIT, children exhibited enhanced bimanual coordination, hand dexterity, and speed of the affected hand use.

## Discussion

The primary purpose of this study was to examine the change over time in real-world bimanual performance using accelerometers in children with UCP after undergoing HABIT. The findings of the study suggest that immediately after HABIT, there were significant improvements in bilateral magnitude, median acceleration, and a trend in improvement in use and magnitude ratios, indicating enhanced bimanual performance. However, during the 3- and 6-month follow-up assessments, the bimanual performance of the children exhibited a progressive decline, nearly returning to baseline values at 6 months following HABIT, as evidenced by the declining numbers in accelerometer-derived variables. Moreover, in line with previous studies, our results demonstrated improvements in UE capacity immediately after HABIT, as reflected by improved scores on standardized assessments. Overall, this is the first study to utilize wearable sensor technology to objectively investigate the change over time in retaining bimanual performance following HABIT. Our results underscore the clinical observation that while real-world bimanual performance may improve after HABIT, bimanual hand use in the real-world context gradually decreases over time in children with UCP.

The primary objective of rehabilitation is to achieve measurable improvements in real-world performance that can be retained over a long period of time. However, so far, none of the studies have examined the retention of UE performance over time post-HABIT, using a reliable and valid tool that accurately reflects real-world performance. In our study, we aimed to address this knowledge gap by utilizing accelerometer-derived variables. Altogether, our findings reveal a progressive decline in these variables following the immediate post-HABIT gains, evident at the 3- and 6-month time period. Immediately following HABIT, we observed improvements or a trend in improvement in real-world bimanual performance, reflected by changes in accelerometer-derived variables. Specifically, variables such as use ratio, magnitude ratio, bilateral magnitude, median acceleration, and acceleration variability showed improvements of 5.5%, 31.3%, 14.9%, 32.9%, and 8.2%, respectively, compared to baseline. These findings indicate greater contributions of the more affected UE to real-world bimanual activities in terms of hours of use (UR) and magnitude (MR, range of movement), enhanced overall magnitude (BM) by both UEs, and greater speed (MA) and variability (AV) of the more affected UE movements. Our results contradict previous studies by Goodwin et al., [29] and Coker-Bolt et al., [28] which utilized accelerometers to examine UE performance immediately post-CIMT in children with CP. The limited gains reported in their studies could be attributed to the inherent nature of the CIMT approach, which lacks a bimanual training component. We believe, the intense, functionally oriented bimanual tasks practice employed in our HABIT protocol may have been sufficient to surpass a threshold, resulting in notable improvements in bimanual capacity that translated to enhanced real-world performance [37, 38]. Additionally, the varied contextual demands of the bimanual tasks used in this study may have facilitated the transfer of skills and automaticity to real-life bimanual activities.

During follow-up assessments, we observed some variability in the declining trends among the accelerometer-derived variables. Gains achieved in the use ratio immediately post-HABIT showed a slight reduction at 3 months by 0.7%, indicating retention of the immediate gains in terms of duration of use of the more affected UE relative to the less affected UE. Subsequently, however, these improvements showed a substantial decrease of 4.7% at 6 months, almost nearing baseline levels. Similar trends were observed in magnitude ratio, with a 16.7% decrease at the 3-month time-point, indicating some retention of improvements in the more affected UE’s magnitude towards real-world performance. Subsequently, these gains were lost (42.6%), falling back closer to baseline levels at 6-month time point. Overall, the findings suggest a decline in the contribution of the more affected UE to daily tasks, indicating reduced bimanual performance in terms of hours and movement amplitude of the affected UE, potentially leading to a greater contribution of the less affected UE to bimanual tasks. On the contrary, BM (which represents the overall magnitude of movements), MA (mean acceleration of the more affected UE), and AV (variability of the more affected UE) showed different trends compared to UR and MR. Our results showed a remarkable decrease in BM (12.3%), MA (17.7%), and AV (4.1%) at the 3-month follow-up assessment, followed by further slight reduction at 6 months post-HABIT. These findings suggest that total magnitude of both UEs, and the accelerations and variability in movements of the more affected UE decline considerably within the first 3 months post-HABIT, indicating no retention of performance. This disparity in trends among the accelerometer variables may indicate that the decline in real-world performance might be slower for variables such as use and magnitude ratios, depicting the bimanual coordination aspect of daily tasks. We postulate that the retention of performance observed 3 months following HABIT in UR and MR could be due to the better transfer of skills that were trained during our focused, task-specific, bimanual training during HABIT. However, the lack of supervised, structured practice outside the HABIT could have led to the deterioration of the performance gains over a 6-month period. In contrast, the decrease in the performance of the more affected UE (median acceleration and acceleration variability) and overall magnitude of movements (bilateral magnitude) is faster following the immediate gains, suggesting lack of retention even until 3 months. MA and AV represent the mean acceleration and the variability in accelerations performed by the more affected UE. We believe that during HABIT, the more affected UE was involved in intensive bimanual and gradually progressive complex bimanual activities. The lack of such task-specific practice in daily life may have resulted in limited retention at 3-month time point. Moreover, our HABIT protocol also consisted of several overhead and large range of movement tasks such as shooting a basketball, playing tennis, hitting a baseball, or playing corn hole etc. Limited practice of similar activities following the intensive bouts of training could have resulted in the rapid decline on the overall magnitude of movements (BM) at the 3-month follow-up.

Previously, only one study utilized accelerometers to examine the long-term effects of UE task-specific practice on upper extremity (UE) performance in individuals with stroke [27]. The study findings indicate no changes in accelerometer derived variables over time following an 8-week task-specific UE training in post-stroke individuals. It was posited that the chronicity of stroke, motivational, and environmental factors may have contributed to the limited performance gains. Importantly, our findings contradict previous studies that support long-term retention of bimanual performance post-intensive training in children with UCP [19, 39, 40]. These studies utilized standardized assessments such as Assisting Hand Assessment and three-dimensional motion analysis to assess hand function and movement efficiency. However, these standard tools only measure UE capacity, reflecting what a child does in a controlled environment. Moreover, UE performance was assessed using subjective measures such as Canadian Occupational Performance measure, which reflect the perception of changes in the real-world performance by either the parent or child and is prone to biases [21]. Accelerometer metrics used in our study overcome these limitations and depict real-world hand use in children with UCP. Additionally, the studies supporting long-term retention using standardized assessments involved home programs after the end of the trial, which may have contributed to the observed long-term retention of capacity measures [18, 20]. Our study did not involve any home exercise program, potentially impacting the long-term retention. Altogether, this study is unique in three ways: 1) it is the first time accelerometers were used as a tool to examine retention of bimanual performance over time following HABIT in children with UCP, 2) we demonstrated that, despite undergoing HABIT, the real-world performance of children with UCP returns to baseline levels within 6 months, and 3) the information gathered in our study could be valuable for future researchers and clinicians, in determining the critical periods to re-intervene and prevent further decline in upper extremity function in children with UCP.

Our results also demonstrated significant improvements in upper extremity capacity immediately post-HABIT, as reflected by changes in the scores of standardized clinical tests. Notably, post-HABIT, the AHA score exceeded the minimal clinically important difference of 5 logit units [33] suggesting enhanced affected hand function and bimanual coordination. In addition, children completed the NHPT (18.7% faster) and JHFT (14.3% faster) quicker, indicating greater speed and dexterity post-HABIT. These findings are consistent with those reported by Gordon et al., [41] which emphasizes the role of the intensity of therapy in enhancing hand capacity. Similar findings of improvement in hand capacity have been reported by Sakzewski et al., [39] and Facchin et al. [40] In our study, we believe the intensive practice of goal-oriented tasks may have contributed to improvements in manual dexterity and speed, reiterating improvements in capacity measures of the more affected hand following HABIT in children with UCP.

We acknowledge a few limitations in our study and propose directions for future research. First, the absence of a control group in our study limits the explicit interpretation of the results. Including a control group in the future studies would help in confidently determining whether the changes in outcomes are due to the intervention or to some other variable. Second, due to the feasibility issues, we were unable to examine changes in bimanual capacity measures during the 3- and 6-month follow-up assessments. It would have been interesting to observe parallel changes in the UE capacity over time, and this aspect should be considered in future research. Third, the lack additional assessments time-points between post-training and 3 months, and between 3 and 6 months. The inclusion of these time-points in future studies could provide accurate insights into the temporal patterns of retention of bimanual performance. Fourth, the findings of our study should be interpreted with caution, as accelerometers are known to capture both purposeful and non-purposeful movements, such as arm swings during walking. Thus, a portion of our data may include these non-purposeful movements. However, some previous studies have shown alignment between accelerometer-recorded activity counts and purposeful repetitions observed in therapy for the affected UE [42, 43]. Lastly, the generalizability of our study may be limited due to the heterogeneity of the population.

## Conclusions

HABIT appears to enhance real-world bimanual performance and capacity immediately post-training as measured by accelerometer-derived variables and standardized assessments. However, these improvements gradually decline within 3 months post-HABIT, with a near-complete regression to pre-training levels within 6 months after HABIT, indicating a time-dependent deterioration of real-world bimanual function. Our findings hold potential clinical and research implications, suggesting the need for careful consideration of the critical time for re-intervention to mitigate further decline of bimanual function and maintain the quality of life in children with UCP.

## Supporting information

S1 Data (XLSX)
